# Family history of malignant or benign thyroid tumors: implications for surgical procedure management and disease-free survival

**DOI:** 10.3389/fendo.2023.1282088

**Published:** 2023-11-29

**Authors:** Yu-jia Jiang, Zhuo-jun Han, Yi-xuan Hu, Ning Zhang, Tao Huang

**Affiliations:** Department of Breast and Thyroid Surgery, Union Hospital, Tongji Medical College, Huazhong University of Science and Technology, Wuhan, China

**Keywords:** benign thyroid neoplasms, thyroid nodules, thyroid cancer, familial nonmedullary thyroid carcinoma, family history, surgery benign thyroid neoplasms, disease-free survival

## Abstract

**Background:**

Current guidelines lack a standardized management for patients with family history of thyroid carcinoma (fTC),particularly benign thyroid neoplasm (fBTN). Our objective was to investigate the influence of various family histories on the selection of surgical approaches and disease-free survival (DFS).

**Methods:**

A cohort study was conducted involving 2261 patients diagnosed with differentiated thyroid carcinoma including those with fTC (n=224), fBTN (n=122), and individuals without a family history of thyroid carcinoma (nfTC; n=1915). Clinicopathological characteristics were collected. DFS was estimated using Kaplan-Meier analysis and factors affecting DFS were identified using Cox proportional hazard model.

**Results:**

Compared to nfTC, small tumor size, clinically lymph node-positive, extrathyroidal extension, vascular invasion, Hashimoto’s disease and nodular goiter were more common in fTC and fBTN groups. They had lower T stage and a lower rate of good response to TSH suppression therapy but received more radioiodine therapy. It is worth noting that fTC is associated with male, bilateral and multifocal tumors, as well as central lymph node metastasis and distant metastasis. Both fTC (aHR = 2.45, 95% CI=1.11-5.38; *P* = 0.03) and fBTN (aHR = 3.43, 95% CI=1.27-9.29; *P* = 0.02) were independent predictors of DFS in patients who underwent lobectomy, but not total thyroidectomy. For 1-4 cm thyroid carcinomas with clinically node-negative, fTC was identified as an independent predictor, whereas fBTN was not.

**Conclusion:**

Our findings indicate that a family history, particularly of malignancy, is associated with a more aggressive disease. Family history does not affect the prognosis of patients who undergo total thyroidectomy, but it may increase the risk of postoperative malignant events in those who have a lobectomy. Additionally, it may be necessary to monitor individuals with a family history of benign thyroid neoplasms.

## 1 Introduction

Currently, the most contentious issue regarding family history of thyroid carcinoma is familial non-medullary thyroid carcinoma (FNMTC), which accounts for approximately 5-10% of all cases of thyroid carcinoma (1). FNMTC is characterized by the presence of two or more first-degree relatives affected by non-medullary thyroid carcinoma. However, current studies have not yet reached a definitive consensus on whether FNMTC tumors exhibit distinct biological behavior and prognosis compared to sporadic non-medullary thyroid carcinoma (SNMTC) in individuals (2–9).

Moreover, thyroid neoplasms can be benign or malignant, and it is unclear if individuals with a family history of benign thyroid neoplasm (BTN) should receive the same level of attention as those with FNMTC. Studies in Asian populations have shown a coaggregation of chronic thyroid diseases and multiple malignancies with non-medullary thyroid carcinoma (NMTC) (10). Other studies in Kuwait, Caledonia, and Iran found an association between a family history of benign thyroid disease (BTD) and an increased risk of thyroid cancer, indicating a familial susceptibility (11–13). However, D. Kust et al. (14) argued that a family history of BTD does not lead to the development of thyroid cancer. It is worth noting that papillary thyroid carcinoma (PTC) in children and adolescents has been linked not only to a family history of thyroid carcinoma but also to a family history of BTD (15).

In light of the current controversy surrounding family history, our study aims to analyze the characteristics as well as prognosis of patients with a family history of thyroid neoplasm. We will focus on assessing the effects of family history on the treatment and prognosis of patients with thyroid cancer, particularly those in the clinically lymph node-negative (cN0) stage with 1-4 cm tumors.

## 2 Methods

### 2.1 Patients and data

We obtained data from our department’s clinical database, which included a total of 2261 patients from January 2010 to May 2022. Patients were directly asked about their first-degree relatives (parents, offspring, and siblings) who had been diagnosed with thyroid cancer or other thyroid diseases, and this information was documented in their medical records. First-degree relatives with suspicious nodules underwent confirmation through pathological examination. Based on hospital records, patients were classified into three groups: Patients without a family history were defined as non-familial cases (nfTC). Additionally, patients with a family history of thyroid neoplasm were categorized into two groups: fTC for those with a family history of malignancy, and fBTN for individuals with a family history of benign cases, including nodular goiter.

Patients with medullary thyroid carcinoma, undifferentiated thyroid cancer, a family history of nonthyroidal malignant tumors, or those who underwent head and neck radiotherapy were excluded from the study. Ultimately, we included 1915 nfTCs, 224 fTCs, and 112 fBTNs in this study. PTC accounts for 99% in the group without a family history, 99.1% in the group with a family history of thyroid cancer, and 100% in the group with a family history of benign thyroid tumors.

Among families with two affected first-degree relatives, we included 137 patients in the fTC group and 50 patients in the fBTN group. Additionally, there were 87 patients from families with three or more affected first-degree relatives in the fTC group and 72 patients in the fBTN group. The distribution of patients in terms of belonging to the parental generation or offspring, as well as the classification of inheritance patterns as either horizontal or vertical, is presented in **Table 1**.

**Table 1:** Patient characteristics^a^.

Characteristics	nfTC (n=1915)	fTC (n=224)	*P*_1_ ^c^	fBTN (n=122)	*P*_2_ ^c^
Male	467 (24.4)	69 (30.8)	**0.04**	28 (23.0)	0.72
Age, median (IQR), y	44.0 (34.0-50.6)	43.0 (33.0-51.0)	0.43	41.0 (33.0-50.0)	0.14
≤ 55	1632 (85.2)	190 (84.8)	0.87	108 (88.5)	0.32
Family history					
The number of patients in the family with PTC					
2	NA^b^	137 (61.2)	NA	50 (41.0)	NA
≥ 3	NA	87 (38.8)		72 (59.0)	
Generation					
1^st^	NA	103 (46.0)	NA	58 (47.5)	NA
2^nd^	NA	121 (54.0)		64 (52.5)	
Genetic Mode					
sibling	NA	59 (26.3)	NA	38 (31.1)	NA
parent-offspring	NA	165 (73.7)		84 (68.9)	

nfTC, without a family history of thyroid carcinoma; fTC, family history of thyroid carcinoma; fBTN, family history of benign thyroid neoplasm; PTC, Papillary thyroid carcinoma; NA, not available.

a Chi-square test or Fisher’s exact chi-square test or Mann-Whitney U test; Data are given as number (percentage) of patients unless otherwise specified.

b Data were not available due to no family history.

c The *P*-value that holds statistical significance is highlighted in bold.

### 2.2 Management and follow-up

We followed guideline-based approaches to select different surgical strategies, including total thyroidectomy (TTD) or lobe thyroidectomy (LTD). Based on preoperative ultrasound or imaging evaluation, prophylactic unilateral/bilateral central lymph node dissection (CLND) was performed for patients with cN0 stage, while therapeutic central/lateral lymph node dissection (LLND) was conducted for those with clinical lymph nodes-positive (cN1). According to the risk stratification of patients, they received serum thyroid-stimulating hormone (TSH) suppression therapy and radioiodine (RAI) therapy.

Our department routinely performs follow-up including ultrasonography and serum thyroid function tests. Follow-up is conducted at 1, 3, and 6 months after surgery, with subsequent follow-up every 6-12 months if no suspicious lesions are detected. Additional imaging tests such as neck magnetic resonance imaging, chest computed tomography, or 18F-fluorodeoxyglucose positron emission tomography may be done if there are concerns about local recurrence or distant metastasis. Recurrence or metastasis is confirmed through fine needle aspiration cytology or core needle biopsy.

Follow-up data included disease-free survival (DFS), which was defined as the time from tumor-free status until biopsy or serial imaging confirmation of residual thyroid recurrence (RTR), lymph node metastasis (LNM), or distant metastasis (DM).

### 2.3 Clinicopathological features

We collected information, including basic details, family history, ultrasound evaluation, and treatment strategies. Postoperative pathological parameters included the pathological type, tumor location, number, presence of Hashimoto’s disease or nodular goiter, overall diameter (OD), maximum diameter (MaxD), central lymph node metastasis (CLNM), lateral lymph node metastasis (LLNM), intraglandular dissemination (ITM), extrathyroidal extension (ETE), and vascular infiltration (VI). OD was assessed by summing the maximum diameter of each lesion. Multifocality is defined as having more than one lesion on a single-sided thyroid gland. In this study, if there was one lesion on each side (bilateral), it was not considered multifocal. Postoperative treatments encompassed TSH suppression therapy, reoperation, and radioiodine (RAI) therapy. The American Joint Committee on Cancer (AJCC) tumor-node-metastasis (TNM) staging system was used to classify thyroid carcinoma, and all parameters were evaluated according to the World Health Organization criteria.

### 2.4 Statistical analysis

Statistical analyses were performed using AKIBM SPSS 22.0 was used (IBM Corp., Armonk, NY, USA), and GraphPad Software 8.0.1 (GraphPad Software Inc., San Diego, CA, USA) was used to create figures. Non-normally distributed continuous variables were presented as median (interquartile range [IQR]) and compared using the Mann-Whitney U test. To compare categorical variables between two groups, we used Chi-square and Fisher’s exact tests (Although this study involved multiple groups, the statistical analysis was conducted by comparing the study group to the same control group, without involving multiple comparisons), and we noted group fTC vs. nfTC as *P*_1_, while fBTN vs. nfTC was noted as *P*_2_. The Kaplan-Meier curve, Gehan-Breslow-Wilcoxon and log-rank test were employed to assess the impact of individual variables on prognosis. We utilized a Cox proportional hazards regression model (Cox-model) to identify independent risk factors. The model included adjusted hazard ratio (aHR), 95% confidence interval (CI), and *P*-values for the variables. All reported *P* values in this study were two-sided, and we considered *P* values ≤ 0.05 to be statistically significant.

## 3 Results

### 3.1 Patients’ characteristics

**Table 1** displays the demographic characteristics of the study participants. It is worth noting the proportion of male patients was significantly higher in the family history of thyroid carcinoma group (30.8%) compared to the non-family history group (24.4%) (*P* = 0.04). However, no significant differences were observed among patients age ≤ 55 years.

### 3.2 Preoperative evaluation

Patients with a family history of malignant tumors exhibited a higher prevalence of bilateral nodules (56.3% vs. 48.0%) and multifocality(70.1% vs. 50.8%) compared to patients without a family history (all *P* < 0.05).

The MaxD/OD sizes were significantly smaller in the family histories groups (all *P* < 0.001). Moreover, cN1 was more commonly observed in both the family history of malignant tumor group (19.6%) and the benign tumor group (19.7%), (all *P* < 0.001) (**Table 2**).

**Table 2:** Preoperative ultrasound characteristics of the patients^a^.

Preoperative ultrasound	nfTC (n=1915)	fTC (n=224)	*P*_1_ ^b^	fBTN (n=122)	*P*_2_ ^b^
Bilaterality	919 (48.0)	126 (56.3)	**0.02**	61 (50.0)	0.67
Isthmus nodule	197 (10.3)	27 (12.1)	0.41	13 (10.7)	0.90
Multifocality	972 (50.8)	157 (70.1)	**<0.001**	71 (58.2)	0.11
MaxD, median (IQR), cm	1.1 (0.8-1.8)	0.9 (0.5-1.5)	**<0.001**	0.5 (0.2-1.2)	**<0.001**
1 to 4	1078 (56.3)	91 (40.6)	**<0.001**	42 (34.4)	**<0.001**
OD, median (IQR), cm	1.8 (1.1-2.8)	1.4 (0.7-2.1)	**<0.001**	0.8 (0.3-1.6)	**<0.001**
1 to 4	1330 (69.5)	126 (56.2)	**<0.001**	44 (36.1)	**<0.001**
Clinical N stage					
clinically node-negative	1761 (92.0)	180 (80.4)	**<0.001**	98 (80.3)	**<0.001**
clinically node-positive	154 (8.0)	44 (19.6)		24 (19.7)	

nfTC, without a family history of thyroid carcinoma; fTC, family history of thyroid carcinoma; fBTN, family history of benign thyroid neoplasm; MaxD, maximum diameter; OD, overall diameter.

a Chi-square test or Fisher’s exact chi-square test or Mann-Whitney U test; Data are given as number (percentage) of patients unless otherwise specified.

b The *P*-value that holds statistical significance is highlighted in bold.

### 3.3 Treatment and postoperative pathology

The selection of surgical approaches did not show significant differences between the family history groups and the non-family history group (all *P* > 0.05) according to **Table 3**.

**Table 3:** Management of the patient’s surgical approach^a^.

Treatment	nfTC (n=1915)	fTC (n=224)	*P*_1_ ^b^	fBTN (n=122)	*P*_2_ ^b^
Total thyroidectomy	1568 (81.9)	172 (76.8)	0.06	99 (81.1)	0.83
Lobe thyroidectomy	347 (18.1)	52 (23.2)		23 (18.9)	
Central lymph node dissection	1859 (97.1)	214 (95.5)	0.21	116 (95.1)	0.33
Lateral lymph node dissection	392 (20.5)	48 (21.4)	0.74	25 (20.5)	>0.99

nfTC, without a family history of thyroid carcinoma; fTC, family history of thyroid carcinoma; fBTN, family history of benign thyroid neoplasm.

a Chi-square test or Fisher’s exact chi-square test or Mann-Whitney U test; Data are given as number (percentage) of patients unless otherwise specified.

b The *P*-value that holds statistical significance is highlighted in bold.

In our study, PTC was the most common pathology type. The size of tumors and the presence of multifocal bilateral thyroid carcinomas demonstrated a consistent trend with preoperative ultrasound findings.

There were significant differences in the occurrence of extrathyroidal extension and vascular invasion between the familial groups and the non-familial group (all *P* < 0.001). In the familial thyroid cancer group, 62.1% of patients experienced central lymph node metastasis, which was higher compared to non-familial group (52.2%) and the family history of benign thyroid nodules group (55.7%). However,there was no significant difference in lateral lymph node metastasis. Additionally, the occurrence of Hashimoto’s disease and nodular goiter was higher in both the familial thyroid cancer and familial benign thyroid nodules groups (all *P* < 0.001).

As shown in **Table 4**, the proportions of T stage in family history of thyroid carcinoma and benign nodule were 75.0% and 79.6%, respectively. These proportions were lower than the non-familial group (90.4%). Significant differences were also noted in the M stage (*P* = 0.02), but not in the N stage (*P* > 0.05), between the group with a family history of thyroid cancer and non-familial group.

**Table 4:** Postoperative pathological characteristics of the patients^a^.

Postoperative pathology	nfTC (n=1915)	fTC (n=224)	*P*_1_ ^b^	fBTN (n=122)	*P*_2_ ^b^
Tumor type					
Papillary thyroid carcinoma	1896 (99.0)	222 (99.1)	> 0.99	122 (100)	0.63
Follicular thyroid carcinoma	19 (1.0)	2 (0.9)		0 (0.0)	
Multifocality	828 (43.2)	114 (50.9)	**0.03**	56 (45.9)	0.57
Bilaterality	609 (31.8)	86 (38.4)	**0.04**	40 (32.8)	0.82
MaxD, median (IQR), cm	0.7 (0.5-1.2)	0.5 (0.2-1.0)	**<0.001**	0.4 (0.2-0.9)	**<0.001**
1 to 4	712 (37.2)	61 (27.2)	**0.01**	27 (22.1)	**0.001**
OD, median (IQR), cm	1.0 (0.5-1.5)	0.7 (0.3-1.5)	**<0.001**	0.4 (0.2-1.1)	**<0.001**
1 to 4	917 (47.9)	78 (34.8)	**0.001**	33 (27.1)	**<0.001**
Intraglandular dissemination	91 (4.8)	10 (4.5)	0.85	5 (4.1)	0.74
Extrathyroidal extension	64 (3.3)	30 (13.4)	**<0.001**	20 (16.4)	**<0.001**
Vascular infiltration	6 (0.3)	6 (2.7)	**<0.001**	5 (4.1)	**<0.001**
Central lymph node metastasis	999 (52.2)	139 (62.1)	**0.005**	68 (55.7)	0.45
Lateral lymph node metastasis	305 (15.9)	42 (18.8)	0.28	20 (16.4)	0.90
Hashimoto’s disease	325 (17.0)	76 (33.9)	**<0.001**	40 (32.8)	**<0.001**
Nodular goiter	328 (17.1)	66 (29.5)	**<0.001**	36 (29.5)	**<0.001**
T stage					
T:1	1731 (90.4)	168 (75.0)	**<0.001**	97 (79.6)	**<0.001**
T:2	106 (5.5)	14 (6.3)		7 (5.7)	
T:3	67 (3.5)	25 (11.2)		12 (9.8)	
T:4	11 (0.6)	17 (7.5)		6 (4.9)	
N stage					
N0	884 (46.2)	90 (40.2)	0.10	50 (41.0)	0.27
N1	1031 (53.8)	92 (59.8)		72 (59.0)	
M stage					
M0	1900 (99.2)	218 (97.3)	**0.02**	120 (98.4)	0.62
M1	15 (0.8)	6 (2.7)		2 (1.6)	
AJCC					
I	1785 (93.2)	206 (92.0)	**0.06**	117 (95.9)	0.47
II	126 (6.6)	15 (6.7)		5 (4.1)	
III	2 (0.1)	1 (0.4)		0 (0.0)	
IV	2 (0.1)	2 (0.9)		0 (0.0)	

nfTC, without a family history of thyroid carcinoma; fTC, family history of thyroid carcinoma; fBTN, family history of benign thyroid neoplasm; MaxD, maximum diameter; OD, overall diameter.

a Chi-square test or Fisher’s exact chi-square test or Mann-Whitney U test; Data are given as number (percentage) of patients unless otherwise specified.

b The *P*-value that holds statistical significance is highlighted in bold.

### 3.4 Postoperative treatments

Significant differences were observed in the response to TSH suppression therapy (all *P* < 0.001). Specifically, a higher proportion of patients in the non-family history group (73.2%) achieved a good response compared to the family history of thyroid cancer group (67.8%) and the family history of benign thyroid nodules group (61.5%). The most notable differences were found in the category of “Uncertain response,” with proportions of 0.5%, 6.7%, and 17.2% in the respective groups.

The reoperation rate was 2.3%, 8.5%, and 4.9% among patients, with a significant difference only observed between the non-family history group and the family history of thyroid cancer group (*P* < 0.001). Furthermore, 32.5%, 41.1%, and 48.4% of patients received RAI 131 treatment, with significant differences between the non-family history group and family histories groups (*P*_1 = _0.01 and *P*_2_ < 0.001, respectively) (**Table 5**).

**Table 5:** Postoperative follow-up management^a^.

Follow-up	nfTC (n=1915)	fTC (n=224)	*P*_1_ ^b^	fBTN (n=122)	*P*_2_ ^b^
TSH inhibition therapy					
Good response	1403 (73.2)	152 (67.8)	**<0.00**1	75 (61.5)	**<0.001**
Biochemical abnormality	63 (3.3)	12 (5.4)		6 (4.9)	
Structural abnormality	440 (23.0)	45 (20.1)		20 (16.4)	
Uncertain response	9 (0.5)	15 (6.7)		21 (17.2)	
Reoperation	45 (2.3)	19 (8.5)	**<0.001**	6 (4.9)	0.14
Radioiodine therapy	622 (32.5)	92 (41.1)	**0.01**	59 (48.4)	**<0.001**
Recurrence & Metastasis			**<0.001**		0.34
Residual thyroid recurrence	20 (1.0)	7 (3.1)		2 (1.6)	
Lymph node metastasis	85 (4.4)	24 (10.7)		8 (6.6)	
Distant metastasis	15 (0.8)	6 (2.7)		2 (1.6)	
Total follow-up period, median (IQR), months	49 (33–68)	51 (23–79)	0.81	51 (42–61)	0.89

nfTC, without a family history of thyroid carcinoma; fTC, family history of thyroid carcinoma; fBTN, family history of benign thyroid neoplasm.

a Chi-square test or Fisher’s exact chi-square test or Mann-Whitney U test; Data are given as number (percentage) of patients unless otherwise specified.

b The *P*-value that holds statistical significance is highlighted in bold.

### 3.5 Follow-up

The total follow-up duration (median, [IQR], months) were 49 (33–68), 51 (23–79), and 51 (42–61), respectively, with no significant difference. (All *P* > 0.05).

The rate of disease recurrence or metastasis was significantly higher in the familial thyroid cancer than in non-familial group, while there was no significant difference seen in the family history of benign thyroid nodule group (*P*_1_ < 0.001 and *P*_2_ = 0.34, respectively): Specifically, after primary surgery, other lobe recurrence occurred in 1.0%, 3.1%, and 1.6% of patients in the nfTC, fTC, and fBTN groups, respectively. Lymph node metastasis occurred in 4.4%, 10.7%, and 6.6% of patients in those respective groups. Distant metastasis happened in 15 (10 lung metastases, 4 bone metastases, and 1 liver metastasis.), 6 (4 lung metastases and 2 lung-to-brain metastases.), and 2 (2 lung metastases) patients, respectively (**Table 5**).

### 3.6 Effect of family history on the prognosis (DFS) for patients underwent lobectomy or total thyroidectomy

**Figure 1:**
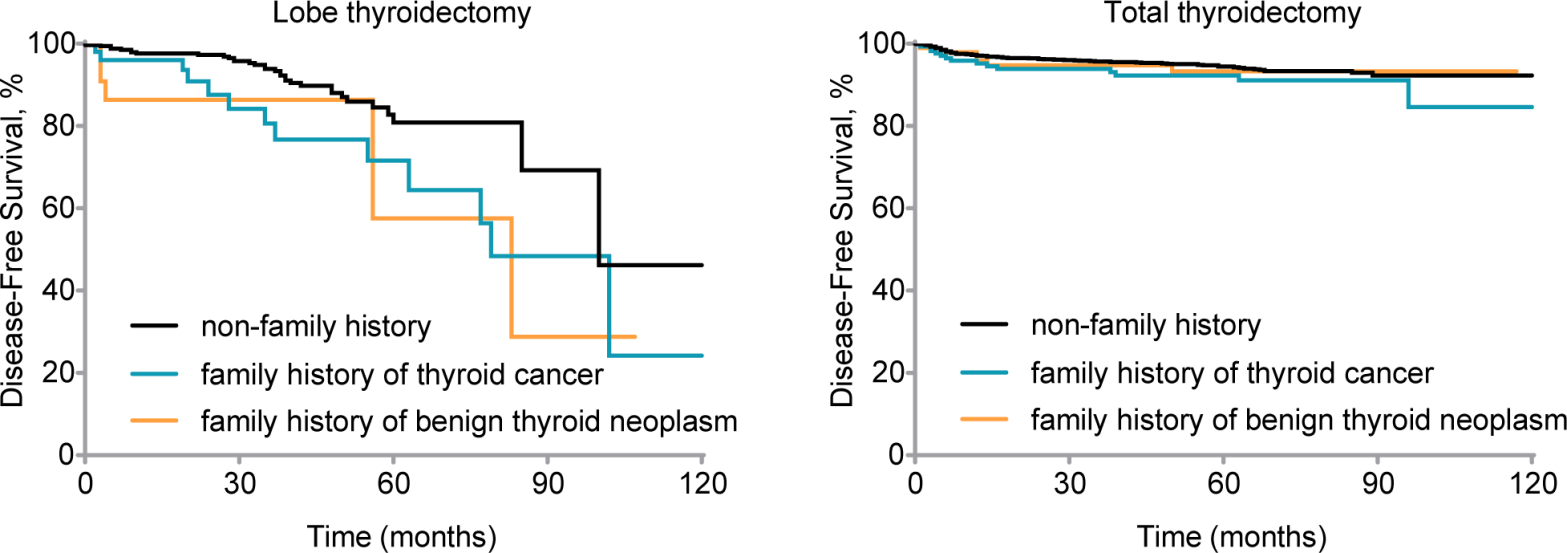
Disease-free survival (DFS) by family history for patients with different treatments. Kaplan-Meier curve displays DFS by different family histories for patients who underwent lobe thyroidectomy (Gehan-Breslow-Wilcoxon test, *P*_1_ = 0.008, *P*_2_ = 0.04; log-rank test, *P*_1_ = 0.02, *P*_2_ = 0.10) and total thyroidectomy (Gehan-Breslow-Wilcoxon test, *P*_1_ = 0.14, *P*_2_ = 0.64; log-rank test, *P*_1_ =0.13, *P*_2_ = 0.72). nfTC, without a family history of thyroid carcinoma; fTC, family history of thyroid carcinoma; fBTN, family history of benign thyroid neoplasms.

We further investigated the prognosis of different family history groups under different surgical approaches. Among patients who underwent lobectomy, the familial thyroid cancer group exhibited a survival difference compared to the group without a family history of thyroid cancer (Gehan-Breslow-Wilcoxon test, *P*_1_ = 0.008; log-rank test, *P*_1_ = 0.02, respectively). On the other hand, the familial benign thyroid nodules group showed a significant difference only in early stages, with no significant difference observed in long-term outcomes. (Gehan-Breslow-Wilcoxon test, *P*_2_ = 0.04; log-rank test, *P*_2_ = 0.10, respectively). For patients who underwent total thyroidectomy, no significant difference in survival was observed between the group with a family history (either of thyroid cancer or benign thyroid nodules) and the group without a family history (all *P* > 0.05) (**Figure 1**).

Compared to the lobectomy, the total thyroidectomy showed a survival advantage: in the lobectomy group, 89.6% of patients without a family history were in a disease-free survival state. However, the disease-free survival state was limited to a mere 67.3% within the group of malignant family history, while the benign family history group account for 78.3%. In the total thyroidectomy group, the disease-free survival rates were 94.6% (no family history), 91.3% (malignant family history), and 93.9% (benign family history) respectively.

Additionally, in the lobectomy group, the median survival time was shorter in the family history group compared to the non-family history group: 100 months (non-family history group), 79 months (malignant family history), and 83 months (benign family history). Patients with a family history of thyroid cancer had and other lobe recurrence rate of 13.5%, whereas those with a family history of benign thyroid nodules had a rate of 8.7%. In contrast, the other lobe recurrence rate was only 5.8% in patients without a family history. Furthermore, postoperative lymph node metastasis occurred in 15.4% of patients with a family history of thyroid cancer and 13.0% of patients with a family history of benign thyroid nodules in the familial group. In comparison, only 4.0% of patients without a family history experienced lymph node metastasis. Distant metastasis was observed in 3.8% of patients with a family history of thyroid cancer, while it occurred in only 0.6% of patients without a family history.

**Figure 2:**
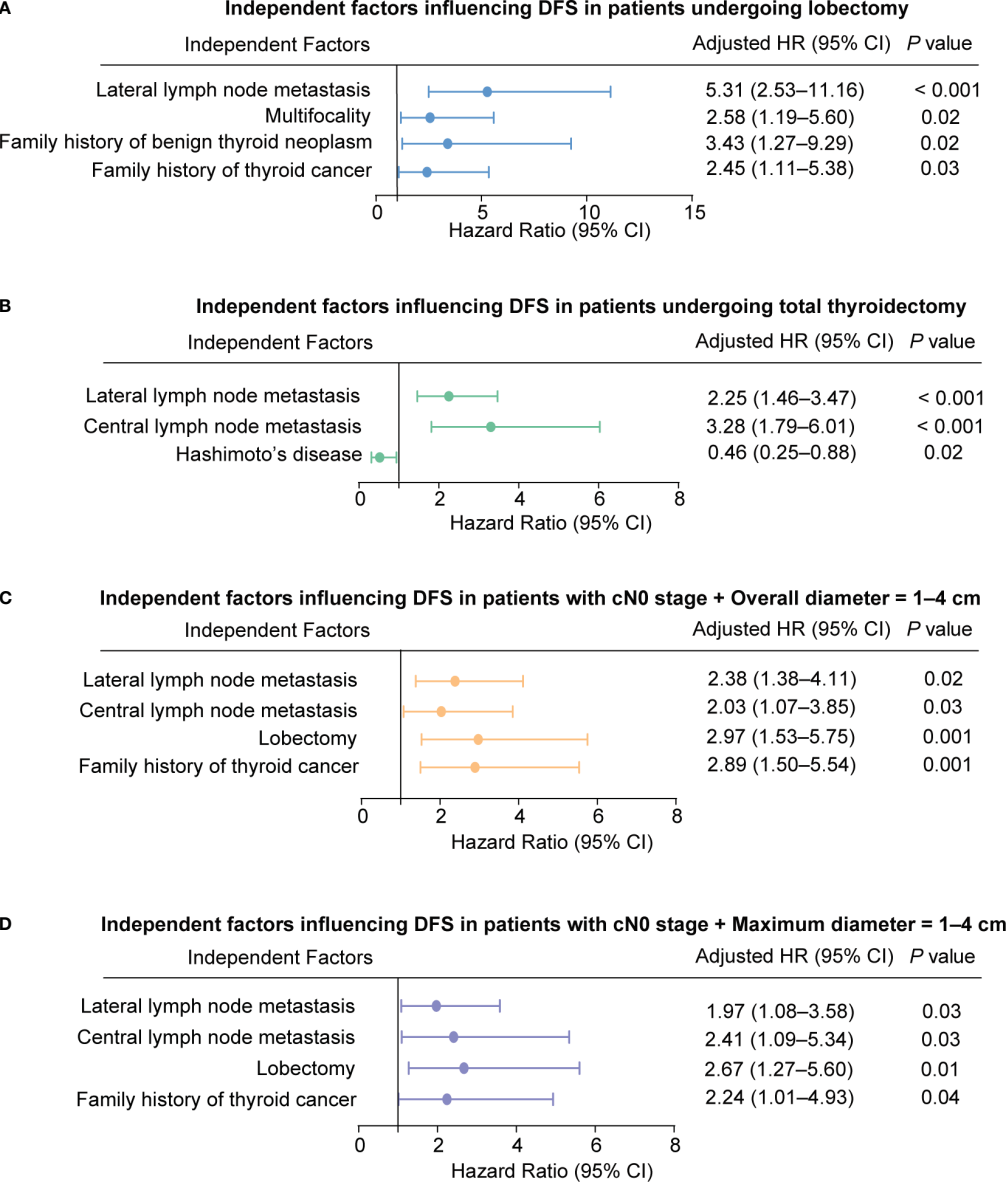
Independent factors influencing Disease-Free Survival (DFS) in Patients. **(A)** Patients undergoing lobectomy. **(B)** Patients undergoing total thyroidectomy. **(C)** Patients with cN0 stage and overall diameter of 1-4 cm. **(D)** Patients with cN0 stage and maximum diameter of 1-4 cm.

We then included all parameters in the multivariable Cox-model (**Table 6**). The results showed that for the lobectomy group, a family history of thyroid cancer (aHR = 2.45, 95% CI = 1.11–5.38; *P* = 0.03), a family history of benign thyroid nodules (aHR = 3.43, 95% CI = 1.27–9.29; *P* = 0.02), Multifocality (aHR = 2.58, 95% CI = 1.19–5.60; *P* = 0.02) and lateral lymph node metastasis (aHR = 5.31, 95% CI = 2.53–11.16; *P* < 0.001) were independent risk factors that affected DFS (**Figure 2A**). In the total thyroidectomy group, family history was not an independent factor (all *P* > 0.05). Hashimoto’s disease (aHR = 0.46, 95% CI = 0.25–0.88; *P* = 0.02), central lymph node metastasis (aHR = 3.28, 95% CI = 1.79–6.01; *P* < 0.001), and lateral lymph node metastasis (aHR = 2.25, 95% CI = 1.46–3.47; *P* < 0.001) were predictive independent factors of prognosis (**Figure 2B**).

**Table 6:** Multivariable Cox Proportional Hazards Regression Model for Disease-Free Survival (DFS).

Predictors	Lobe thyroidectomy		Total thyroidectomy		cN0+OD 1-4 cm		cN0+MaxD 1-4 cm	
	aHR (95% CI)	*P* Value^a^	aHR (95% CI)	*P* Value^a^	aHR (95% CI)	*P* Value^a^	aHR (95% CI)	*P* Value^a^
Sex								
Male	1[Reference]		1[Reference]		1[Reference]		1[Reference]	
Female	0.81 (0.43-1.64)	0.61	1.11 (0.72-1.71)	0.64	1.21 (0.69-2.15)	0.50	1.15 (0.62-2.12)	0.66
Age								
≤55	1[Reference]		1[Reference]		1[Reference]		1[Reference]	
>55	1.18(0.51-2.76)	0.69	0.84 (0.45-1.60)	0.60	1.53 (0.77-3.04)	0.23	1.17 (0.52-2.64)	0.70
Family history								
nfTC	1[Reference]		1[Reference]		1[Reference]		1[Reference]	
fTC	2.45 (1.11-5.38)	**0.03**	1.59 (0.88-2.89)	0.13	2.89 (1.50-5.54)	**0.001**	2.24 (1.01-4.93)	**0.04**
fBTN	3.43 (1.27-9.29)	**0.02**	1.54 (0.64-3.69)	0.34	0.87 (0.12-6.40)	0.89	1.17 (0.16-8.70)	0.88
Treatment								
Total thyroidectomy	NA	NA	NA	NA	1[Reference]		1[Reference]	
Lobe thyroidectomy	NA	NA	NA	NA	2.97 (1.53-5.75)	**0.001**	2.67(1.27-5.60)	**0.01**
Multifocality								
No	1[Reference]		1[Reference]		1[Reference]		1[Reference]	
Yes	2.58 (1.19-5.60)	**0.02**	1.18 (0.61-2.28)	0.62	1.32(0.62-2.83)	0.48	1.53 (0.66-3.53)	0.32
Location								
Laterality	1[Reference]		1[Reference]		1[Reference]		1[Reference]	
Bilaterality	0.56 (0.17-1.90)	0.35	1.39 (0.75-2.60)	0.30	0.99 (0.47-2.11)	0.98	0.85 (0.36-2.01)	0.71
MaxD								
< 1	1[Reference]		1[Reference]		NA	NA	NA	NA
1 to 4	1.18 (0.30-4.71)	0.81	1.13 (0.60-2.13)	0.70	NA	NA	NA	NA
≥4	0.53 (0.02-12.0)	0.69	3.02 (0.70-13.03)	0.14	NA	NA	NA	NA
OD								
< 1	1[Reference]		1[Reference]		NA	NA	NA	NA
1 to 4	1.17 (0.31-4.44)	0.81	1.38 (0.69-2.80)	0.37	NA	NA	NA	NA
≥4	2.30 (0.15-34.26)	0.55	1.69 (0.47-6.11)	0.43	NA	NA	NA	NA
Intraglandular dissemination								
No	1[Reference]		1[Reference]		1[Reference]		1[Reference]	
Yes	0.66(0.19-2.36)	0.52	1.63 (0.92-2.90)	0.10	0.81 (0.28-2.33)	0.70	0.60(0.14-2.49)	0.48
Extrathyroidal extension								
No	1[Reference]		1[Reference]		1[Reference]		1[Reference]	
Yes	0.57 (0.15-2.19)	0.41	1.63 (0.92-2.90)	0.12	1.50 (0.70-3.20)	0.29	1.93 (0.87-4.26)	0.10
Vascular infiltration								
o	1[Reference]		1[Reference]		1[Reference]		1[Reference]	
Yes	2.27 (0.28-18.47)	0.44	0.36 (0.05-2.80)	0.33	1.31 (0.17-10.19)	0.80	0.68 (0.08-5.59)	0.72
Hashimoto’s disease								
No	1[Reference]		1[Reference]		1[Reference]		1[Reference]	
Yes	0.75 (0.37-1.55)	0.44	0.46 (0.25-0.88)	**0.02**	0.58 (0.27-1.23)	0.15	0.59 (0.25-1.43)	0.59
Nodular goiter								
No	1[Reference]		1[Reference]		1[Reference]		1[Reference]	
Yes	1.85 (0.83-4.13)	0.13	0.96 (0.55-1.70)	0.90	0.84 (0.40-1.78)	0.65	1.15 (0.50-2.64)	0.75
Central lymph node metastasis								
No	1[Reference]		1[Reference]		1[Reference]		1[Reference]	
Yes	0.80 (0.42-1.53)	0.51	3.28 (1.79-6.01)	**<0.001**	2.03 (1.07-3.85)	**0.03**	2.41 (1.09-5.34)	**0.03**
Lateral lymph node metastasis								
No	1[Reference]		1[Reference]		1[Reference]		1[Reference]	
Yes	5.31 (2.53-11.16)	**<0.001**	2.25 (1.46-3.47)	**<0.001**	2.38 (1.38-4.11)	**0.02**	1.97 (1.08-3.58)	**0.03**

cN0, clinical node-negative; nfTC, without a family history of thyroid carcinoma; fTC, family history of thyroid carcinoma; fBTN, family history of benign thyroid neoplasm; MaxD, maximum diameter; NA, not available.

a The *P*-value that holds statistical significance is highlighted in bold.

### 3.7 Effect of family history on the prognosis (DFS) for patients with cN0 stage thyroid carcinoma measuring 1–4 cm

**Figure 3:**
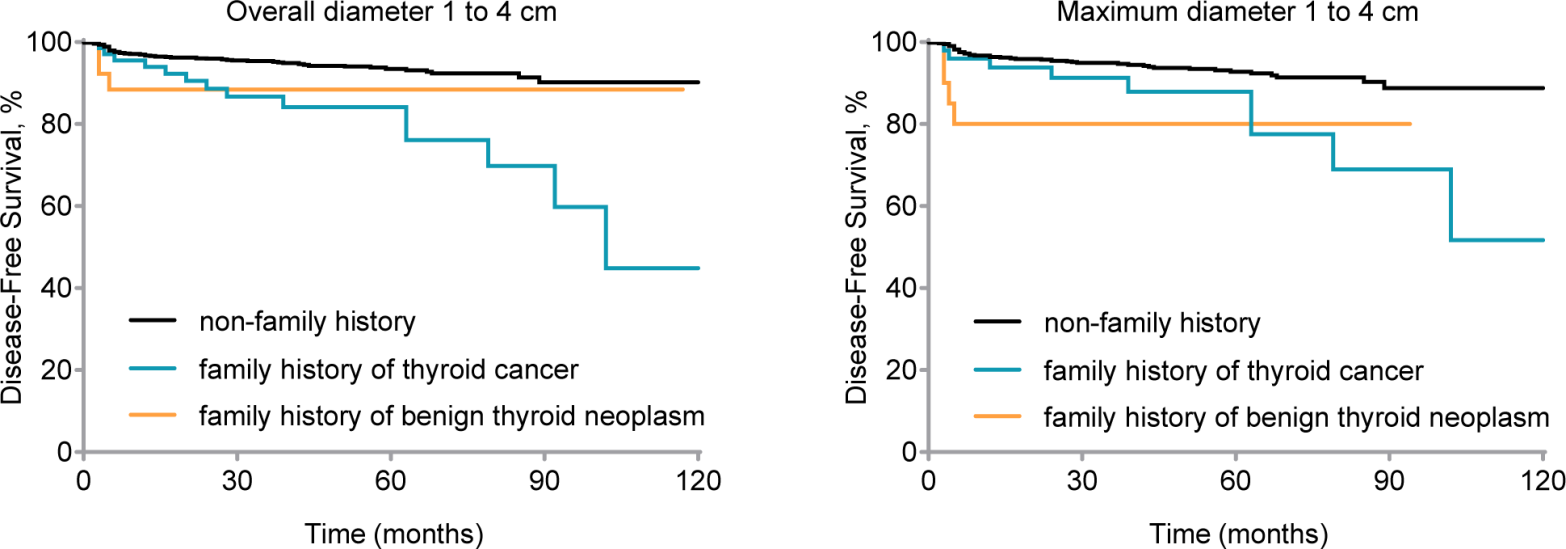
Disease-free survival (DFS) by family history for clinical node-negative (cN0) stage patients with 1 to 4 cm thyroid carcinoma. Kaplan-Meier curve displays DFS by different family histories for patients of cN0 stage and overall diameter 1 to 4 cm thyroid carcinoma (Gehan-Breslow-Wilcoxon test, *P*_1_ = 0.001, *P*_2_ = 0.11; log-rank test, *P*_1_ < 0.001, *P*_2_ = 0.23) and maximum diameter 1 to 4 cm thyroid carcinoma (Gehan-Breslow-Wilcoxon test, *P*_1_ = 0.08, *P*_2_ = 0.002; log-rank test, *P*_1_ = .004, *P*_2_ = 0.01). nfTC, without a family history of thyroid carcinoma; fTC, family history of thyroid carcinoma; fBTN, family history of benign thyroid neoplasms.

We conducted a study on thyroid carcinomas measuring 1–4 cm, which has been controversial in cN0 stage. We used the MaxD and OD as measures of thyroid cancer size and performed survival analysis for OD = 1–4 cm (Gehan-Breslow-Wilcoxon test, *P*_1_ = 0.001, *P*_2_ = 0.11; log-rank test, *P*_1_ < 0.001, *P*_2_ = 0.23, respectively) and MaxD of 1–4 cm (Gehan-Breslow-Wilcoxon test; *P*_1_ = 0.08, *P*_2_ = 0.002, log-rank test, *P*_1_ = .004, *P*_2_ = 0.01; respectively) (see **Figure 3**).

According to **Table 6**, a family history of thyroid cancer (aHR = 2.89, 95% CI = 1.50–5.54; *P* = 0.001), undergoing lobectomy (aHR = 2.97, 95% CI = 1.53–5.75; *P* = 0.001), having pathologically confirmed central lymph node metastasis (aHR = 2.03, 95% CI = 1.07–3.85; *P* = 0.03), and having lateral lymph node metastasis (aHR = 2.38, 95% CI = 1.38–4.11; *P* = 0.02) were all identified as independent risk factors affecting DFS in patients with cN0 stage and tumor size (Overall diameter) between 1 and 4 cm (**Figure 2C**).

Furthermore, similar findings were observed for MaxD as for OD. A family history of thyroid cancer (aHR = 2.24, 95% CI = 1.01–4.93; *P* = 0.04), undergoing lobectomy (aHR = 2.67, 95% CI = 1.27–5.60; *P* = 0.01), having pathologically confirmed central lymph node metastasis (aHR = 2.41, 95% CI = 1.09–5.34; *P* = 0.03), and having lateral lymph node metastasis (aHR = 1.97, 95% CI = 1.08–3.58; *P* = 0.03) were identified as independent risk factors affecting prognosis (**Figure 2D**).

## 4 Conclusion

This article primarily focuses on investigating the clinicopathological features and evaluating the influence of surgical methods on the prognosis of patients with a family history of thyroid neoplasms, encompassing both malignant and benign cases.

Some studies have shown differences in aggressive behavior, recurrence, and survival between FNMTC and SNMTC (5, 9, 16–18), while others have not (6, 7, 19–22). The controversy surrounding FNMTC may stem from different definitions and interpretations. Some argue that a significant portion of 2-hits families may represent incidental occurrences rather than genetic susceptibility (22), while others suggest that familial cases should be linked to affected generations and the number of affected individuals (23–31). Nonetheless, FNMTC has been observed to occur at an earlier age and exhibit more aggressive features in offspring, supporting its classification as a true familial disease even when only two family members are affected (32). In addition, since susceptibility genes for FNMTC have not been identified and there is genetic heterogeneity (33–35), it is considered unreliable to exclude the possibility of FNMTC when only two first-degree relatives are affected.

Current research has been discussing whether having a family history of BTD increases the likelihood of developing thyroid cancer (10–15). In clinical practice, it is not uncommon to see patients with a family history of benign thyroid nodules. From a genetic perspective (33–35), it is possible that thyroid carcinoma is a genomically incomplete epiphenomenon, meaning that family members may possess a susceptibility gene for thyroid carcinoma but exhibit only benign nodules or remain in a healthy state. Therefore, it is necessary to investigate the family history of BTN when evaluating the risk of developing thyroid cancer.

The study examined a group with a family history of benign thyroid tumors and found several clinical features compared to the non-family history group. These included smaller tumor diameter, a higher detection rate of cN1, a higher likelihood of ETE and VI, and a higher association with Hashimoto’s disease and nodular goiter. The proportion of T1 stage tumors was lower. Additionally, they had a higher rate of radioiodine therapy and a lower rate of good response to TSH suppression therapy. These findings may suggest that this particular patient population could potentially have more severe disease.

These findings were similar to the characteristics observed in fTC. However, compared to the non-family history group, fTC also showed a greater tendency for male predominance, higher rates of bilateral and multifocal tumors, an elevated incidence of CLNM and distant metastasis. Additionally, fTC carried a higher risk of reoperation and recurrence after primary surgery.

The preceding discussion primarily centered around the static disease characteristics observed in patients with a family history. However, the dynamic prognosis is contingent upon two factors: the selection of the initial surgical approach and the nature of the disease itself. Our results indicate that there was no difference in prognosis between familial and non-familial types of patients who underwent total thyroidectomy. However, among the familial thyroid cancer group, patients who underwent lobectomy exhibited a survival diaparity compared to non-familial group. As for the group with benign thyroid nodules, there was a significant difference only in the early stages, but no significant difference in long-term outcomes.

Additionally, a family history of malignant or benign thyroid tumors is an independent risk factor that affected the prognosis of patients with lobectomy but not total thyroidectomy. This, on the other hand, indicates that the prognosis of patients undergoing total thyroidectomy is not strongly associated with a family history background. Incomplete surgery may be the cause of these differences, as seen in the fBTN group. Survival analysis showed a higher occurrence of malignant events in patients with a family history of benign thyroid nodules during the early postoperative period, indicating that physicians who did not consider the patient’s family history may have performed incomplete surgery.

The impact of family history on the treatment for 1-4 cm thyroid carcinoma in the cN0 stage remains unclear (36, 37). In our study, we reassessed MaxD and OD as criteria and concluded that a malignant family history was an independent risk factor for the prognosis of OD or MaxD = 1-4 cm tumors. Conversely, a benign family history was not found to be an independent risk factor for 1-4 cm tumors.

Given that patients may not always have full awareness of their own condition or the extent of the disease within their families, our aim was to provide a simple and straightforward reference value by considering only the presence of a family history, rather than relying on complex predictive models. Based on our findings, we recommend that patients with a family history (either benign thyroid nodules or thyroid carcinoma), particularly in cases of multifocality, undergo total thyroidectomy in order to avoid postoperative malignant events. Additionally, a family history of thyroid carcinoma, can serve as an independent prognostic factor for stage cN0 with 1-4 cm thyroid cancer, providing guidance in selecting the surgical approach.

The study had several limitations, including the need to expand the study population. Moreover, we plan to continue patient follow-up with an expected median follow-up of 10 years. Additionally, it is important to note that prognosis is influenced not only by clinical features but also by susceptibility genes, other cancers, environmental factors, and more. Currently, the treatment for thyroid cancer tends to be conservative, advocating for active surveillance (38, 39). However, if the presence of a family history is due to genetic alterations that contribute to disease development, such as the well-known BRAF mutation, even patients with microcarcinomas may be considered at intermediate-to-high risk (39). Therefore, when facing patients with a family history, it is unreasonable to solely rely on general characteristics of thyroid cancer to assess their actual condition. These aspects should be considered in future studies.

## Data availability statement

The raw data supporting the conclusions of this article will be made available by the authors, without undue reservation.

## Ethics statement

The study was approved by Union Hospital Tongji Medical College Huazhong University of Science and Technology Ethics Committee (2017-S062). The studies were conducted in accordance with the local legislation and institutional requirements. Written informed consent for participation was not required from the participants or the participants’ legal guardians/next of kin because This is a retrospective study based on clinical database review.

## Author contributions

Y-JJ: Conceptualization, Data curation, Formal analysis, Funding acquisition, Writing – original draft. Z-JH: Data curation, Formal analysis, Writing – original draft. Y-XH: Data curation, Writing – original draft. NZ: Methodology, Supervision, Writing – review & editing. TH: Conceptualization, Funding acquisition, Supervision, Writing – review & editing.
